# A Robust Algorithm for Optimisation and Customisation of Fractal Dimensions of Time Series Modified by Nonlinearly Scaling Their Time Derivatives: Mathematical Theory and Practical Applications

**DOI:** 10.1155/2013/178476

**Published:** 2013-09-17

**Authors:** Franz Konstantin Fuss

**Affiliations:** School of Aerospace, Mechanical and Manufacturing Engineering, RMIT University, Bundoora Campus, Plenty Road, P.O. Box 71, Bundoora, Melbourne VIC 3083, Australia

## Abstract

Standard methods for computing the fractal dimensions of time series are usually tested with continuous nowhere differentiable functions, but not benchmarked with actual signals. Therefore they can produce opposite results in extreme signals. These methods also use different scaling methods, that is, different amplitude multipliers, which makes it difficult to compare fractal dimensions obtained from different methods. The purpose of this research was to develop an optimisation method that computes the fractal dimension of a normalised (dimensionless) and modified time series signal with a robust algorithm and a running average method, and that maximises the difference between two fractal dimensions, for example, a minimum and a maximum one. The signal is modified by transforming its amplitude by a multiplier, which has a non-linear effect on the signal's time derivative. The optimisation method identifies the optimal multiplier of the normalised amplitude for targeted decision making based on fractal dimensions. The optimisation method provides an additional filter effect and makes the fractal dimensions less noisy. The method is exemplified by, and explained with, different signals, such as human movement, EEG, and acoustic signals.

## 1. Introduction

The Hausdorff-Besicovitch dimension, *D*_*H*_, is defined by most efficient covering [1] of irregular curves and surface profiles, usually approximated by the box-counting, circle-counting, or yardstick methods. In these methods, an irregular curve is covered by *N* number of boxes of size *r*, circles of radius *r*, or rulers of length *r*. The Hausdorff dimension, *D*_*H*_, is then determined from

(1) $$\begin{array}{c} D_{H}=\underset{r\to 0}{\lim}\frac{\log N}{\log\left(1/r\right)}. \end{array}$$

This method dates back to Felix Hausdorff who coined the term “fractal dimension” (“gebrochene Dimension,” [2]) by extending Carathéodory's [3] *p*-dimensional measure to noninteger values of *p*. It was reinvented by Richardson [4], for investigating the complexity and ruggedness of coastlines with yardstick methods, who empirically found the following equation:

(2) $$\begin{array}{c} \Sigma l\propto l^{-\alpha }, \end{array}$$

where *l* is the length of the yardstick (e.g., in km), Σ*l* is the sum of all yardsticks covering the total length, and the exponent *α* characterises the irregularity of the coastline or the frontier. It was Mandelbrot [5] who recognised that this exponent corresponds to a fractal dimension, to be calculated from the gradient of log⁡*N* against log⁡1/*r*.

Rewriting Richardson's equation (2) in the form of

(3) $$\begin{array}{c} L_{r}=\lambda r^{1-D}, \end{array}$$

where *L* is the length of a curve (a coastline or frontier in Richardson's cases), *λ* is a constant, and *D* = *D*_*H*_. Considering that *L* = *Nr*, (3) implies that

(4) $$\begin{array}{c} N=\lambda {\left(\frac{1}{r}\right)}^{D}, \end{array}$$

(5) $$\begin{array}{c} \log N=\log\lambda +D\log\frac{1}{r}, \end{array}$$

where log⁡*λ* is the intercept and *D* is the gradient when plotting log⁡*N* against log⁡ 1/*r*, or

(6) $$\begin{array}{c} -\frac{\log N}{\log\left(r\right)}=-\frac{\log\lambda }{\log\left(r\right)}+D, \end{array}$$

which shows that plotting −log⁡*N*/log *r* against log⁡*r* delivers a reciprocal function of log *r*, with the asymptotic value of *D*, comparable to finite element convergence tests. In fact, increasing the number of elements *N* results in *r* → 0.

The Hausdorff dimension *D*_*H*_ is therefore conveniently explained as the asymptotic value of the ratio of log *N* to log⁡1/*r* as *r* → 0. The fact that *D* is the exponent in (4) links *D*_*H*_ to the Hurst exponent *H*, considering that *D*_*H*_ = 2 − *H*.

In addition to the standard box-counting, circle-counting, or yardstick methods, more effective methods were developed, such as the methods by Katz [6], Higuchi [7], Sevcik [8], and Raghavendra and Dutt (multiresolution box-counting MRBC and multiresolution length-based MRL methods [9]). The choice of mathematical methods gained fresh momentum when Raghavendra and Dutt [9, 10] compared existing methods and found Katz' method [6] to be highly inaccurate. They also demonstrated the bad correlation of a hypnogram with the corresponding sleep EEG's fractal dimensions calculated with Katz' method, whereas Higuchi's method provided a good correlation [10]. Based on Raghavendra and Dutt's [10] results, Castiglioni [11] investigated Katz' method, identified flaws, and argued that Katz' fractal dimension is “*strongly influenced by amplitude, duration and units of measure of the waveform, resulting practically useless for any real biomedical application*.” As Katz [6] calculates the Euclidean distance between consecutive points, Castiglioni [11] criticises the fact that this method “sums together terms with different units” (e.g., the unit of the time scale such as seconds and the unit of the signal amplitude such as millivolt). Castiglioni [11] suggests an alternative method, based on Mandelbrot's [12] approach that considers a mono-dimensional space, that is, the signal amplitude only, instead of a two-dimensional approach (signal amplitude and time scale). Higuchi's [7] method is also calculated in a monodimensional space. According to Blaszczyk and Klonowski [13], “…*it is important that scaling of the signal amplitude, has no influence on the results, since it causes only parallel shifting of the regression line along the *ln⁡(*L*(*k*))* axis *[annotation: ln⁡(*L*(*k*)) = log⁡*N* in (5)], *without changing its angular coefficient* [annotation: angular coefficient = gradient *D* of (5)].” This refers to the principle of a mono-dimensional space, where only the signal amplitude is considered for calculating the fractal dimension. In contrast to that, scaling of the signal amplitude when using the Euclidean distance affects the fractal dimension (Castiglioni [11]). 

The problem of calculating the length of a signal through the Euclidean distance of amplitude and time dimensions is that quantities of different units, for example, milliVolt (mV) and seconds (s), are squared and summed up, and finally the sum is square rooted. This does not only apply to Katz' [6] method but also to Raghavendra and Dutt's [9] multiresolution length-based MRL method. Moreover, Raghavendra and Dutt's [9] multiresolution box-counting MRBC method calculates the ratio of quantities of different units: the number of boxes required to cover the distance *h* between two consecutive data points is ceil(|*h*|/*dt*), where ceil is the ceiling function and *dt* is the reciprocal value of the sampling frequency. Subsequently, the amplitude of the next data point *y*_(*i*+1)_ is updated by summing up the amplitude of the previous datum *y*_(*i*)_, the distance *h*, and *dt*: *y*_(*i*+1)_ = *y*_(*i*)_ ± |*h*| ∓ *dt*, thereby summing up different units (e.g., mV and s). 

These problems can be overcome when using Sevcik's method [14], by normalising the amplitude of a signal to its range (*y*_max⁡_ − *y*_min⁡_) and the time axis to the duration of the signal (*t*_max⁡_), thereby transforming the signal into a unit square. This procedure solves the problem of confounding different units but renders signals with different data ranges and periods incomparable. If two signals recorded at 100 Hz for periods of 5 s and 10 s are compared, then Δ*x* of the unit square is 0.002 and 0.001, respectively. Assuming the signals have the same *y*_max⁡_ and *y*_min⁡_, then signal 1 appears to be stretched by a factor of 2 in *x*-direction (former time scale) compared to signal 2, that is, the *x*-scale of signal 1 is multiplied by two. The same principle applies to equal period but different data range signals: if the data range of signal 2 is twice the one of signal 1, then Δ*y* of the unit square of signal 1 is twice as large as Δ*y* of signal 2, and the amplitude of signal 1 is multiplied by two (multiplier *m* = 2) compared to signal 2. As already mentioned by Castiglioni [11], scaling of the signal's amplitude, that is, applying different multipliers to *x*- and *y*-axes, results in different fractal dimensions when using the Euclidean distance for calculating the fractal dimensions. Whereas the unit square method appears to be a uniform method across all signals, Sevcik's method in fact applies variable multipliers *m* to signal amplitudes *y*, namely, *m* = *t*_max⁡_/(*y*_max⁡_ − *y*_min⁡_). However, in order to compare the fractal dimensions across signals, the multiplier *m* must be the same, when using the Euclidean distance for calculating the fractal dimensions. In contrast to that, when applying a mono-dimensional space, that is, Higuchi's method, to determining the fractal dimension of a signal, then the multiplier *m* has no effect [13].

The comparability problem when transforming a signal into a unit square could be overcome when using a normalisation factor that is common to all signals that are to be compared in terms of their fractal dimensions. Such normalisation factors could be as follows: (a) the resolution of the recording device (*y*-axis); (b) standard deviation of the signal; and (c) the window width (*x*-axis) used for a running average method. The disadvantage when using the resolution of the recording device is that different research teams might use recording devices with different resolution, or a company releases the next generation of recording devices with better resolution. In these cases, the fractal dimensions of signals are no longer comparable. The disadvantage when using the standard deviation of the signal is that the standard deviation is different in similar signals and even changes within the signal's time series. The disadvantage when using the window width of a running average method is that the optimal window width has to be determined beforehand, for example, from sensitivity analysis, and fractal dimensions of a signal cannot be compared any more when introducing a second factor, the multiplier, together with, and in addition to, the window width. Doubling the window width corresponds to multiplying the *x*-axis by 0.5. The only way of avoiding scaling problems when normalising the signal as explained above and still obtaining a dimensionless number is to normalise the signal to unit amplitude and unit time. In the end, it does not matter which parameter the time series signal is normalised to, be it amplitude range, maximum, or resolution; time period or window width; unit amplitude and unit time; as the whole exercise serves only to transform the signal data into dimensionless values in both amplitude and time dimension. This means that a time series signal, measured in milliVolts (amplitude axis) and seconds (time axis), has its amplitude and time normalised to 1 mV and 1 s, respectively.

The aim of this paper is to test Castiglioni's [11] hypothesis, namely, that scaling of a signal, that is, applying different multipliers to normalised and dimensionless *y*- or *x*-axes, results in different fractal dimensions. Furthermore, it will be shown how scaling of a signal's amplitude affects the Euclidean distance between data points and how this principle modifies the signal when viewed and assessed in a monodimensional space for calculating the modified signal's fractal dimension with Higuchi's method [7]. Finally, it will be explored whether scaling of normalised and dimensionless *y*- or *x*-axes provides an optimisation method for calculating fractal dimensions of the modified signal, which enables better decision making, classification, and quantification of events occurring throughout a time series. If decisions hinge on the fractal dimension of a biomedical signal (e.g., automatic detection of ventricular tachycardia), then the difference in fractal dimension between physiological and pathological signals (or more generally, between signals of different classes) should be as clear as possible. Thus, the question arises, whether we can “design” the fractal dimensions by scaling the amplitude of related signal classes such that the difference in fractal dimensions of the modified signal increases and becomes clearer and more pronounced? It has to be clearly stated at that point that the targeted exploration of the optimisation method for fractal dimensions refers to the fractal dimensions of a modified signal and no longer to the fractal dimensions of the original signal.

## 2. Statement of the Problem

**Figure 1:**
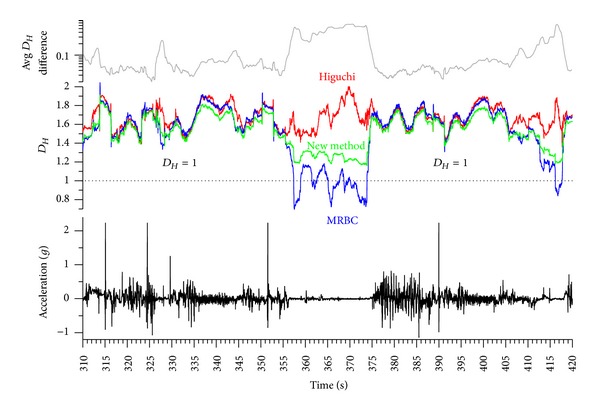
Acceleration of a wheelchair recorded at 60 Hz during a wheelchair rugby match (data from [15]) against time (bottom subfigure); fractal dimension *D*_*H*_ (running average with a window width of 151 data points over 2.5 s) of the acceleration signal calculated with three different methods (middle subfigure): Higuchi's [7] method, Raghavendra and Dutt's [9] MRBC, and the method developed in this study (cf.  Section 4); top subfigure: absolute difference in *D*_*H*_ between the three methods.

Figure 1 shows an acceleration signal and the corresponding *D*_*H*_ calculated with three different methods: Higuchi's method [7], Raghavendra and Dutt's MRBC [9], and the method and algorithm developed in this study. In some parts of the signal, the *D*_*H*_ of all three methods are practically identical in magnitude and shape; in other parts, however, they diverge significantly. Figure 1 serves only the purpose of demonstrating these differences, without making a statement of which method is the best. In fact, as will be shown in Section 7 of this paper, all three *D*_*H*_ are of the same quality and therefore valid and correct (with the exception of *D*_*H*_ < 1). The point here is not the identification of the best method but rather that method which serves best for one's own means, such as for strategic decision making and improved signal classification.

The principle of the MRBC method is to derive the number of boxes covering the signal amplitude change Δ*h* per time step Δ*t* by making the ratio of Δ*h* to Δ*t* integer with a ceiling function and summing up the number of boxes. The principle of Higuchi's method is to sum up the change in amplitude Δ*h* normalised to the time step Δ*t*. This principle is still comparable to box-counting methods, however, by using “boxes” with a “noninteger” height or side length. If a string of data consists only of 0 and 1 (positive and negative), with a device resolution of 1, that is, zero signal with a slight noise, then the number of boxes is zero if two consecutive data are equal. In the MRBC method, relatively small changes in amplitude (with respect to Δ*t*) always deliver a single box. 

The problem for both methods is that a constant signal still has a length—in time direction, but not in amplitude direction. Nevertheless, both methods return zero Δ*h* in such cases. This accounts for zero number of “boxes” (integer or non-integer ones) despite the apparent length of the signal. This problem is not only imminent in longer segments with consecutively identical data, but also influences the *D*_*H*_ if only two consecutive data points are of equal value.

**Figure 2:**
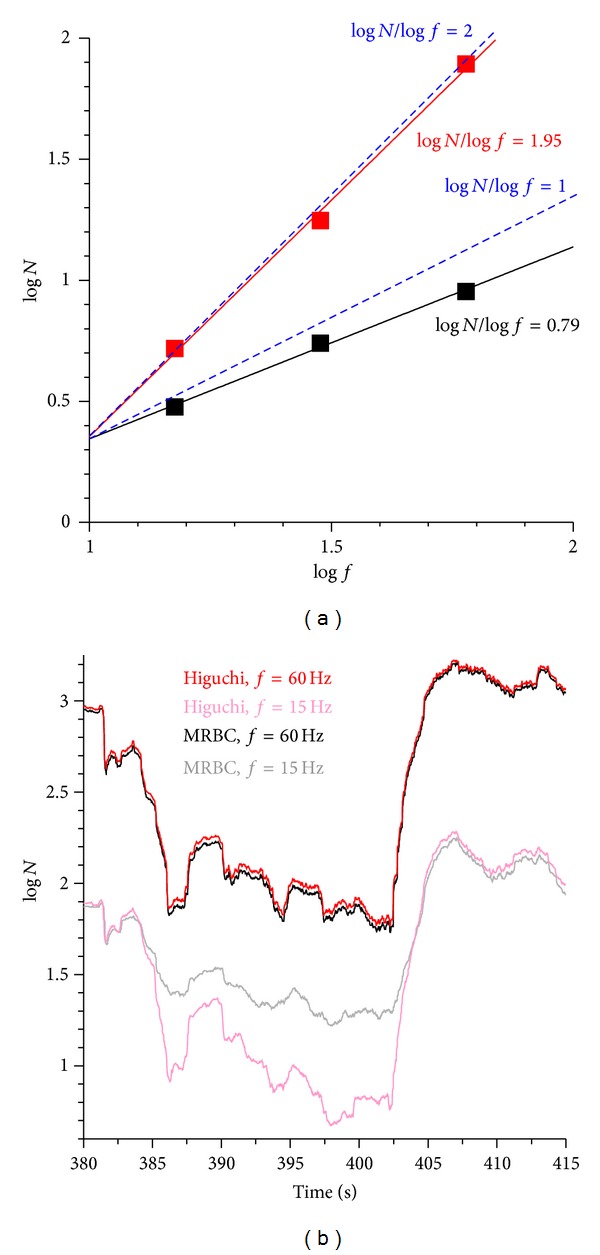
(a) Log *N* against log⁡*f*; *N* = sum  of  amplitudes (Higuchi's method) or boxes (MRBC) of the signal at different frequencies; *f* = 60 Hz (original recording frequency), 30 Hz, and 15 Hz; gradient of *D*_*H*_ = 0.79 (MRBC) was taken at 372.5 s in Figure 1; gradient of *D*_*H*_ = 1.95 (Higuchi's method) was taken at 370 s in Figure 1; (b) log⁡*N* against time (window width = 151 data).

Summing up the number of boxes within a data window at frequencies of *f*, *f*/2, and *f*/4, the gradient of log⁡*N* against log⁡*f* (Figure 2(a)) can be smaller than 1 in the MRBC method and even 2 in Higuchi's method, even at high correlations (*r* > 0.998). It has to be noted that in time series, 1 ≤ *D*_*H*_ ≤ 2. From 369.6 s to 369.95 s in Figure 2, the average difference between Higuchi's method and the MRBC method was 0.969 (Higuchi: 1.986, MRBC: 1.017) with a maximum difference of 0.99 (Higuchi: 2, MRBC: 1.01). It is surprising that two well-performing algorithms deliver opposite dimensions in extreme signals.

**Figure 3:**
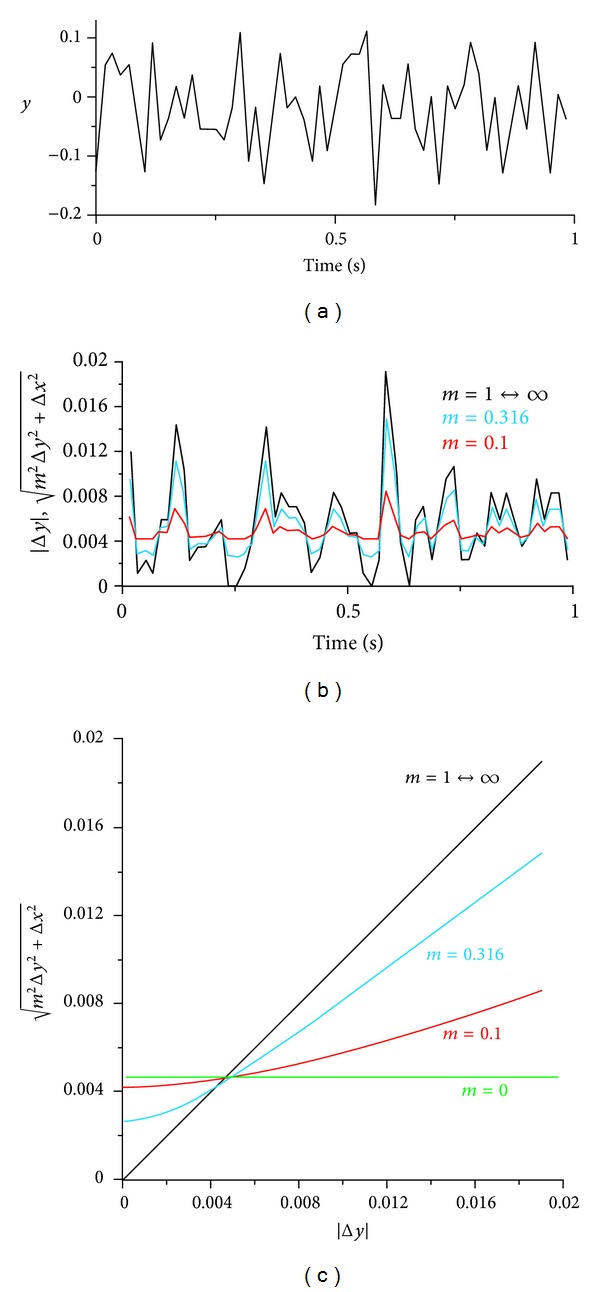
(a) Acceleration signal of Figure 1 from 337 s-338 s; *y* = amplitude; (b) *y*-differential (time derivative of the signal) *L* = |Δ*y*| (Higuchi's method) and Euclidean distance $L=\sqrt{m^{2}\Delta y^{2}+\Delta x^{2}}$ at different *m* against time (*L* is normalised such that the average *L* and the area under the curves are identical in all 3 datasets); (c) Euclidean distance $\sqrt{m^{2}\Delta y^{2}+\Delta x^{2}}$ at different *m* against *y*-differential |Δ*y*|.

The reason for diverging *D*_*H*_ values of Higuchi's method and the MRBC method is explained as follows. If the ratio of Δ*h* to Δ*t* at the original sampling frequency *f* is slightly smaller than 1 (e.g., 0.92), then the number of boxes (*N*) in the MRBC method is 1, whereas the corresponding parameter (*N*) of Higuchi's method remains at 0.92. The ratio of Δ*h* to Δ*t* at *f*/4 is close to 0.23  ( = 0.92/4) in Higuchi's method but still 1 in the MRBC method due to the ceiling function. Logarithming the sum of *N* derived at *f* leads to comparable data with MRBC and Higuchi's methods (Figure 3(b)). However, logarithming the sum of *N* derived at *f*/4 results in far smaller data values when using Higuchi's method than when applying the MRBC method. This is apparent in Figure 2(b) between seconds 385 and 404 where the data derived at *f*/4 diverge significantly. The larger the difference in log⁡*N* data, the steeper the gradient is and thus the larger the fractal dimension is. This explains why Higuchi's method can deliver a fractal dimension of 2, whereas the MRBC method produces a fractal dimension of 1 of the same part of the signal. The ceiling function of the MRBC method leads to different scaling factors at *f*, *f*/2, and *f*/4 if |Δ*h* | /Δ*t* is slightly smaller than 1, which consequently affects the fractal dimension.

Esteller et al. [16] and Raghavendra and Dutt [9, 10] tested different fractal dimension algorithms with *continuous nowhere differentiable functions* (CNDFs, [17, 18]) of known and controllable fractal dimension *D*_*H*_ and with specially designed waveforms (with difference in amplitude and frequency) but not with actual time series signals (biomedical, acoustic, etc.). Such CNDFs are the deterministic Weierstrass cosine, Weierstrass-Mandelbrot cosine, and the Knopp function, as well as the stochastic Brownian motion function. The best performing methods were the Higuchi's method and Raghavendra and Dutt's MRBC and MRL. This proves that these methods are perfectly applicable to the aforementioned CNDFs but not necessarily how well these methods behave in general time series.

## 3. Solution to the Problem

In order to assess the influence of scaling a signal on its fractal dimension *D*_*H*_, *D*_*H*_ has to be determined in a 2-dimensional space rather than in a mono-dimensional one. It is therefore required that the length of a signal is calculated from the Euclidean distance between data points of time series. This makes *D*_*H*_ variable when scaling the amplitude [11], to be shown subsequently. The main question in this case is not “what is the correct method of calculating fractal dimensions?,” but rather “how can we maximise the information to be obtained from fractal dimensions?.” The term “maximising the information” is seen from an engineering point of view, that is, using the difference in *D*_*H*_ of two signals (or parts thereof when using a sliding window method) for practical application, for example, for automated decision making. This would apply to classifying sleep stadia from EEG signals, or identifying arrhythmias from EKG signals, by setting off an alarm at the onset of the latter. 

As 1 ≤ *D*_*H*_ ≤ 2, two dimensions are anyway required, of the same physical quantity and measurement unit, as a *D*_*H*_ of 2 does not apply to a mono-dimensional space. This is achieved when plotting signals on paper or on the screen, and then they become real two-dimensional objects. Coastlines, rivers, and the Brownian motion are 2D curves with the same units in the two orthogonal directions. Time series signals have different units (e.g., mV and s) and therefore have to be transformed into a dimensionless space by normalisation. The difficulties of normalisation were already pointed out in Section 1. Therefore, the normalisation suggested in Section 1 is applied:

(7) $$\begin{array}{c} y=\frac{\text{signal}\;\text{amplitude}\left(\text{e}.\text{g}.\;\;\text{mV}\right)}{\text{unit}\;\text{amplitude}\left(\text{e}.\text{g}.\;\;\text{mV}\right)},\;\;x=\frac{\text{signal}\;\text{period}\left(\text{s}\right)}{\text{unit}\;\text{time}\left(\text{s}\right)}, \end{array}$$

where *y* and *x* are dimensionless and the reciprocal value of *x* equals the dimensionless frequency *f*_0_ = 1/*x*. The suggested normalisation to unit amplitude and unit time serves only for avoiding prescaling of the amplitude, which will be modified subsequently by a multiplier *m*. It is essential that this multiplier is the same across all signals to be compared.

After normalisation, a variable multiplier *m* is applied to the signal's amplitude for scaling purposes and for modifying the signal. As the Euclidean distance *L* between two data points is $\sqrt{\Delta y^{2}+\Delta x^{2}}$ (where *x* and *y* are dimensionless and unitless), it becomes $\sqrt{m^{2}\Delta y^{2}+\Delta x^{2}}$ after applying the multiplier *m*. The smaller *m* is, the more *L* is dominated by Δ*x*, the normalised and dimensionless time of (7). The larger *m* is, the more *L* is dominated by Δ*y*, the normalised and dimensionless signal amplitude of (7). If *m* → *∞*, then *L* → *m*Δ*y*, and the fractal dimension calculated from this modified signal corresponds to Higuchi's fractal dimension of the original signal. As Higuchi's fractal dimension is calculated in a mono-dimensional space, the data differential is |Δ*y*|; the fractal dimension based on which is unaffected by scaling.

In order to demonstrate how the signal is modified by scaling the amplitude with the multiplier *m*, the amplitude differential |Δ*y*| and the Euclidean distance $L=\sqrt{m^{2}\Delta y^{2}+\Delta x^{2}}$ of the signal shown in Figure 3(a) are plotted in Figure 3(b). The difference between amplitude differentials at *m* = *∞* and *m* = 1 is extremely small, resulting in Higuchi's fractal dimension if 1 ≤ *m* ≤ *∞* in this specific part of the signal. When reducing *m*, the normalised *L* (Figure 3(b)) below the average closes in on the normalised average *L* faster than the normalised *L* above the average. This non-linear scaling process is also shown in Figure 3(c). The time derivative of the original signal is therefore non-linearly modified when replacing the amplitude differential by the Euclidean distance. In other words, signal modification by replacing the amplitude differential by the Euclidean distance reduces the difference between mean amplitude differential and differential values below average faster than the difference between differential values above average and mean differential.

If the amplitude multiplier *m* = *∞*, then the Euclidean distance is identical to the scaled amplitude differential |Δ*y*|, and therefore the fractal dimensions of the modified signal are identical to Higuchi's fractal dimensions. If the amplitude differential |Δ*y*| is sufficiently large compared to Δ*x*, then multipliers *m* between infinity and values far smaller than infinity (e.g., 1 in the signal shown in Figure 3(a)) deliver Euclidean distances very close to |Δ*y*|, and therefore fractal dimensions are similar to the original signal. The smaller *m* is, the more the Euclidean distance approaches Δ*x*, and the more the signal is converted to a horizontal line. The resulting fractal dimension of the modified signal is therefore 1 if *m* = 0. Consequently, the fractal dimension *D*_*Hm*_ of the modified signal (at any *m*) ranges between the actual fractal dimension *D*_*Ho*_ of the original signal and unity: 1 < *D*_*Hm*_ < *D*_*Ho*_, where *D*_*Hm*_ applies to 0 < *m* < *∞*, and *D*_*Ho*_ applies to *m* = *∞*, as *D*_*Hm*_ → *D*_*Ho*_ if *m* → *∞*.

Determining and selecting the optimal multiplier *m* depends on the maximal difference between the fractal dimensions of two signals (modified by applying the amplitude multiplier *m*) to be compared for decision making. This optimisation process is explained subsequently, after specifying the algorithm and testing its accuracy with *continuous nowhere differentiable functions* (CNDFs, [17, 18]).

## 4. Robust Algorithm

**Figure 4:**
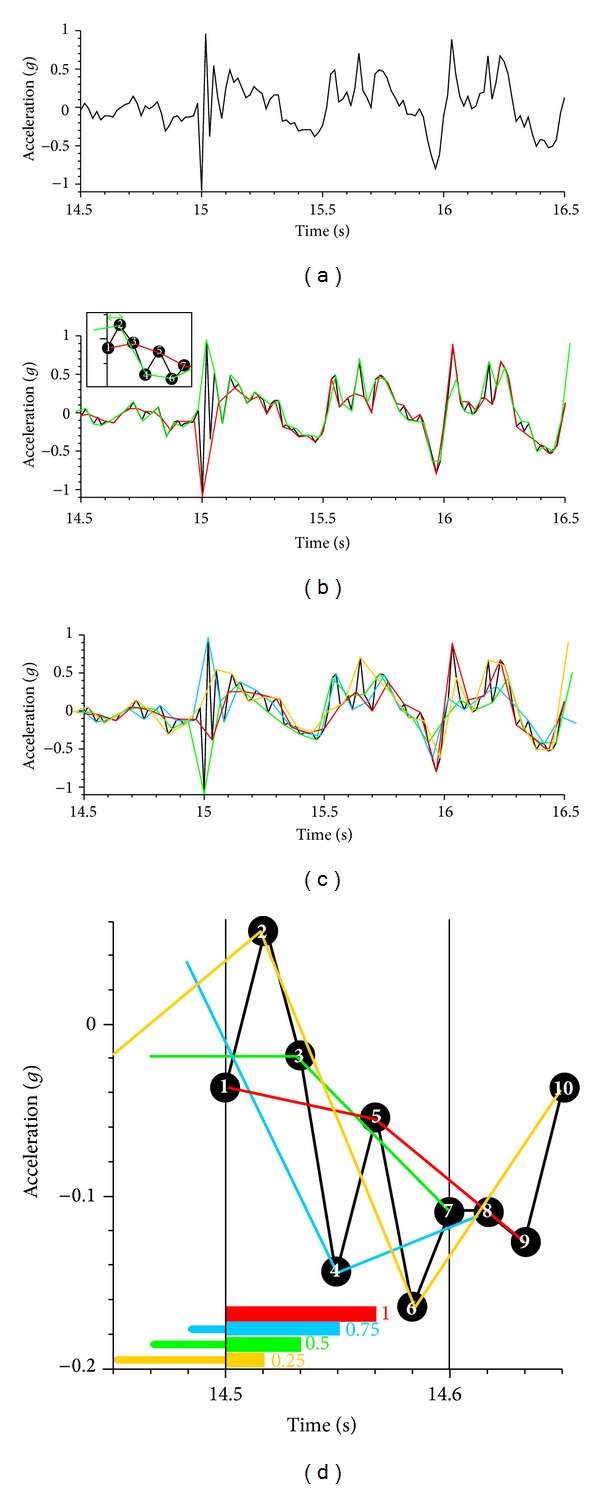
Frequency reduction of a signal; (a) original signal at *f* = 60 Hz; (b) signal reduced to *f* = 30 Hz with two solutions (insert): every other even point (red) and every other odd point (green); insert: ↔ = 2nd half of that green segment which precedes the first green line connecting points 2 and 4; (c) signal reduced to *f* = 15 Hz with four solutions; (d) magnified part of Figure 4(c); the signal starts at 14.5 s; red, yellow, green, and blue lines start at points 1, 2, 3, and 4, respectively; therefore, 1/4, 1/2, and 3/4 of the preceding yellow, green, and blue segments, respectively, have to be included such that each coloured signal starts at 14.5 s exactly.

A signal (Figure 4(a)) with amplitude *y* (dimensionless) is recorded at frequency *f*_0_ (dimensionless) over a window width of *w* data points.

For modifying the signal, the magnitude of the amplitude *y* is multiplied by the multiplier *m*. This method will be subsequently referred to as *modified amplitude fractal dimension method* (MAFDM).

The Euclidean distance *L* between two data points is

(8) $$\begin{array}{c} L_{i}=\sqrt{m^{2}\Delta y^{2}+\Delta x^{2}}=\sqrt{m^{2}{\left(y_{i+1}-y_{i}\right)}^{2}+{\left(\frac{1}{f_{0}}\right)}^{2}}, \end{array}$$

where *y*_*i*_ is the normalised amplitude of an *i*th data point.

The relative length *R* of distance *L* normalised to Δ*x* is

(9) $$\begin{array}{c} R_{i}=\frac{L_{i}}{\Delta x}=f_{0}\sqrt{m^{2}{\left(y_{i+1}-y_{i}\right)}^{2}+{\left(\frac{1}{f_{0}}\right)}^{2}}. \end{array}$$

The relative length over window width *w* starting at datum *i* results from

(10) $$\begin{array}{c} R_{w}=\left(R_{i}+R_{i+1}+\cdots +R_{i+w-1}\right)=\overset{i+w-1}{\underset{i=1}{\sum }}R_{i}. \end{array}$$

Reducing the signal to half *f*_0_ (Figure 4(b)) by taking every other datum yields

(11) Li=m2(yi+2−yi)2+(2f0)2,Ri=f02m2(yi+2−yi)2+(2f0)2.

The relative length over window width *w* starting at datum *i* delivers

(12) Rw =(Ri+Ri+1+⋯+Ri+w−2)+Ri−1(1/2)+Ri+w−2+1(1/2)2 =∑i=1i+w−2Ri+Ri−1(1/2)+Ri+w−2+1(1/2)2.

Equation (12) is divided by two as *R*_*w*_ is the average of two solutions (Figure 4(b)), resulting from taking every other even datum or every other odd datum (insert in Figure 4(b)). Both solutions are embedded in Σ*R*_*i*_ in (12). The terms 0.5*R*_*i*−1_ and 0.5*R*_*i*+*w*−2+1_ are due to the fact that the first green *L* in Figure 4(b) starts only at point no. 2 (insert in Figure 4(b)), and the last green *L* ends at the point preceding the last one of the shown window. This means that half-*L* segments were missing before point 2 (↔ in Figure 4(b)/insert) and after the second last point, if they were not included in the first place.

Reducing the signal to a quarter *f*_0_ (Figure 4(c)) yields

(13) $$\begin{array}{c} L_{i}=\sqrt{m^{2}{\left(y_{i+4}-y_{i}\right)}^{2}+{\left(\frac{4}{f_{0}}\right)}^{2},}\\ R_{i}=\frac{f_{0}}{4}\sqrt{m^{2}{\left(y_{i+4}-y_{i}\right)}^{2}+{\left(\frac{4}{f_{0}}\right)}^{2}.} \end{array}$$

The relative length over window width *w* starting at datum *i* delivers

(14) Rw=((Ri+Ri+1+⋯+Ri+w−4)+Ri−134+Ri−224+Ri−314  +Ri+w−4+134+Ri+w−4+224+Ri+w−4+314)×(4)−1,

(15) Rw=(∑i=1i+w−4Ri+Ri−134+Ri−224+Ri−314  +Ri+w−4+134+Ri+w−4+224+Ri+w−4+314)×(4)−1.

Equation (15) is divided by four as *R*_*w*_ is the average of four solutions (Figure 4(c)). The terms 0.75*R*_*i*−1_, 0.75*R*_*i*+*w*−4+1_, 0.5*R*_*i*−2_, 0.5*R*_*i*+*w*−4+2_, 0.25*R*_*i*−3_, and 0.25*R*_*i*+*w*−4+3_ are required because three-quarters of the preceding blue *L* have to be added to the first blue *L* (which starts at point 4; Figure 4(d)), half of the preceding green *L* has to be added to the first green *L* (which starts at point 3), and a quarter of the preceding yellow *L* has to be added to the first yellow *L* (which starts at point 2, whereas the first red *L* starts at point 2).

Generalising the procedure, with *w* = window width, *j* = start datum of window, and *n* = multiplier of Δ*x* (if *n* = 4, then 4Δ*x* = 4/*f*_0_), *i* and *l* are counters. Note that

(16) Rj,w,n=(∑i=jj+w−nRi+∑l=1nRj−ln−ln  +∑l=1nRj+w−n+ln−ln)×(n)−1=(∑i=jj+w−nRi+∑l=1nn−ln(Rj−l+Rj+w−n+l))(4)−1,

where

(17) $$\begin{array}{c} R_{i}=\frac{f_{0}L_{i}}{n},\\ L_{i}=\sqrt{m^{2}{\left(y_{i+n}-y_{i}\right)}^{2}+{\left(\frac{n}{f_{0}}\right)}^{2}.} \end{array}$$

If *n* = 1, (16) reduces to *R*_*j*,*w*,*n*_ = ∑_*i*=*j*_^*j*+*w*−*n*^ *R*_*i*_, that is, to (10).

It is important to consider equal distribution of *n* on a logarithmic scale, that is, *n* = 1, 2, 4, 8…, or *n* = 1, 3, 9…, and not *n* = 1, 2, 3, 4, 5, as in the latter case *n* is weighted towards smaller frequencies.

The fractal dimension of the modified signal, *D*_*Hm*_, results from

(18) $$\begin{array}{c} \log R_{j,w,n}=D_{Hm}\log\frac{f_{0}}{n}+I, \end{array}$$

where *D*_*Hm*_ is the gradient and *I* is the intercept.

In summary, the new method is characterised by the following:

1. multiplier *m* applied to modifying the signal's amplitude *y*;
2. Euclidean distance *L* between data points;
3. different time resolutions and frequencies (*f*_0_, Δ*x* = original, 2Δ*x* = simulated  half  frequency,…, Δ*x* = 1/*f*_0_);
4. averaging of sum of distances for smaller frequencies (half frequency results in 2 datasets of distances);
5. running the average of *D*_*Hm*_ versus *x*;
6. *D*_*Hm*_ is gradient of −log⁡(Σ*L*_Δ*x*_) versus log⁡(Δ*x*) or log⁡(Σ*L*_Δ*x*_) versus log⁡(*f*_0_) checked by the goodness of fit (correlation coefficient), considering that there are no higher sampling frequencies available other than the original *f*_0_;
7. equidistant lower frequencies such as 1, 2, 4, 8, 16…(not  1, 2, 3, 4, 5…) or 1, 3, 9, 27 … on a log scale.

## 5. Influence of the Signal Amplitude on *D*_*Hm*_

**Figure 5:**
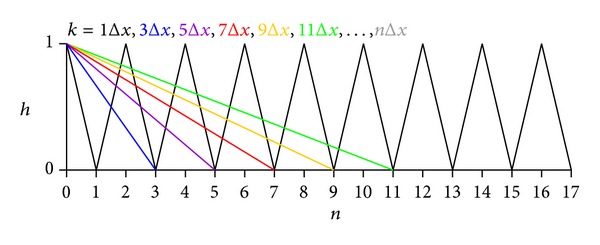
Triangular signal with amplitude *h* against normalised time *x* and normalised time resolution *k*, where *k* = *n*Δ*x*.

In a triangular signal (Figure 5) of constant amplitude *h* (unity) and Δ*x* = 1/*f*_0_, the amplitude *h* is varied by the multiplier *m*.

The time resolution is odd, that is, *k* = 1Δ*x*, 3Δ*x*, 5Δ*x*, 7Δ*x*, 9Δ*x* … *n*Δ*x*.

The length of diagonal at *n* = 3, 5, 7, 9… is

(19) $$\begin{array}{c} L_{k>1\Delta x}=\sqrt{{\left(mh\right)}^{2}+{\left(\frac{n}{f_{0}}\right)}^{2}.} \end{array}$$

The corresponding length of the original signal (*k* = 1Δ*x*) up to *n* is

(20) $$\begin{array}{c} L_{k=1\Delta x}=n\sqrt{{\left(mh\right)}^{2}+{\left(\frac{1}{f_{0}}\right)}^{2}.} \end{array}$$

The square root is multiplied by *n* as the total signal length corresponds to a time period the length of which is *n*Δ*x*. For example, at *n* = 11 (Figure 5), the original triangular signal (*k* = 1Δ*x*) consists of 11 segments, whereas the green diagonal (*k* = 1Δ*x*, Figure 5) consists of a single line. 

The relative length *R* is defined as follows:

(21) $$\begin{array}{c} R_{k>1\Delta x}=\frac{f_{0}}{n}\sqrt{{\left(mh\right)}^{2}+{\left(\frac{n}{f_{0}}\right)}^{2},}\\ R_{k=1\Delta x}=f_{0}n\sqrt{{\left(mh\right)}^{2}+{\left(\frac{1}{f_{0}}\right)}^{2}.} \end{array}$$

The slope *D*_*Hm*_ of a line connecting two points, with the first point defined by relative length *R*_*k*=1Δ*x*_ and frequency *f*_0_ = 1/(1Δ*x*) and the second point defined by *R*_*k*>1Δ*x*_ and *f*_0_ = 1/(*n*Δ*x*) with *n* = 3, 5, 7…, is calculated from

(22) DHm=(log⁡(f0n(mh)2+(1f0)2)  −log⁡(f0n(mh)2+(nf0)2)) ×(log⁡(f0)−log⁡(f0n))−1,

(23) A1,2=lim⁡m→0m→∞((log⁡(f0n(mh)2+(1f0)2)      −log⁡(f0n(mh)2+(nf0)2))     ×(log⁡(f0)−log⁡(f0n))−1),

where *A*_1,2_ are the asymptotic values at *m* → 0 and *m* → *∞*, respectively. Note that

(24) A1,2=lim⁡m→0m→∞(log⁡(f0n(mh)2+(1/f0)2(f0/n)(mh)2+(n/f0)2)     ×(log⁡f0f0/n)−1).

Cancelling out *f*_0_ and calculating each *A* individually,

(25) A1=lim⁡m→0⁡(log⁡(n2(mh)2+(1/f0)2(mh)2+(n/f0)2)     ×(log⁡(n))−1),A1=log⁡(n2((1/f0)2/(n/f0)2))log⁡(n)=log⁡(n2(1/f0)(n/f0))×(log⁡(n))−1=log⁡(n2(f0/f0n))log⁡(n)=log⁡(n)log⁡(n)=1.

This result corresponds to the fractal dimension of a straight line (as *m*, and thus the amplitude, is 0), which has a *D*_*Hm*_ of 1. Note that

(26) A2=lim⁡m→∞(log⁡(n2(mh)2+(1/f0)2(mh)2+(n/f0)2)     ×(log⁡(n))−1),A2=log⁡(n2C)log⁡(n),

where

(27) C=lim⁡m→∞(mh)2+(1/f0)2(mh)2+(n/f0)2=1.

Thus,

(28) $$\begin{array}{c} A_{2}=\frac{\log\left(n^{2}\right)}{\log\left(n\right)}=\frac{2\log n}{\log n}=2. \end{array}$$

This result corresponds to a densely filled area as Δ*x* becomes relatively small with respect to amplitude of *m* → *∞*; the *D*_*Hm*_ of such an area equals 2.

**Figure 6:**
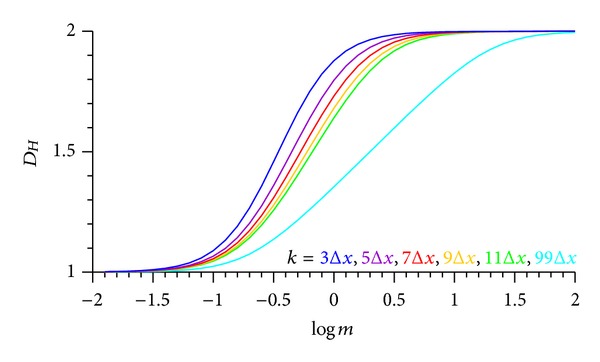
Amplitude spectrum of the fractal dimension *D*_*Hm*_ of the triangular signal of Figure 5 at different time resolutions and different multipliers *m* of the signal amplitude.

If *m* → 0 and *m* → *∞*, then *D*_*Hm*_ asymptotes to 1 and 2, respectively, in this specific triangular signal (Figure 5), the *D*_*Ho*_ of which calculated with Higuchi's method equals 2. Scaling the amplitude via *m* corresponds to stretching or compressing the signal between an area and a straight horizontal line, respectively. This proves that the multiplier *m* of the amplitude decisively influences the signal's fractal dimension which varies between 1 and 2 (Figure 6) in this triangular signal and spans, in general, the range between *D*_*Ho*_ and 1 in different parts of a time series. Figure 6 shows the spectrum of *D*_*Hm*_, calculated from (22), against the multiplier *m*. This spectrum (with a positive gradient) must not be confused with the fractal spectrum (with a negative gradient), calculated from generalised Rényi's entropy (cf. Kulish et al. [19]). The *D*_*Hm*_ spectrum (Figure 6) is decisive for the new (*modified amplitude fractal dimension method*) MAFDM as it enables the selection of *m* for optimisation and customisation purposes, to be explained further later.

## 6. Accuracy of the Proposed Algorithm

The accuracy of the algorithm for the MAFDM, (16)–(18) at *m* = 1, was tested with four CNDFs [17, 18] in order to benchmark it against the performances of Higuchi's and MRBC methods, as reported by Raghavendra and Dutt [9, 10].

(*1) Knopp Function K*(*t*):

(29) $$\begin{array}{c} K\left(t\right)=\overset{\infty }{\underset{k=1}{\sum }}a^{k}\langle \langle b^{k}t\rangle \rangle =\overset{\infty }{\underset{k=1}{\sum }}a^{k}\left|b^{k}t-\left\lfloor b^{k}t+0.5\right\rfloor \right|, \end{array}$$

where 〈〈*x*〉〉 denotes a triangular wave function, that is, the distance from *x* to the nearest integer, and ⌊*x*⌋ denotes the floor function; 0.5 ≤ *a* ≤ 1, *b* > 1, and *ab* ≥ 1.

If 0 ≤ *t* ≤ 1, then the Minkowski-Bouligand dimension *D*_MB_ is

(30) $$\begin{array}{c} D_{\text{MB}}=\frac{\log4a}{\log b}. \end{array}$$

Keeping *b* constant and calculating *a* as a function of *D*_MB_

(31) $$\begin{array}{c} a=\frac{b^{D_{\text{MB}}}}{4}. \end{array}$$

Thus,

(32) $$\begin{array}{c} K\left(t,D_{\text{MB}}\right)=\overset{\infty }{\underset{k=1}{\sum }}{\left(\frac{b^{D_{\text{MB}}}}{4}\right)}^{k}\langle \langle b^{k}t\rangle \rangle . \end{array}$$

**Figure 7:**
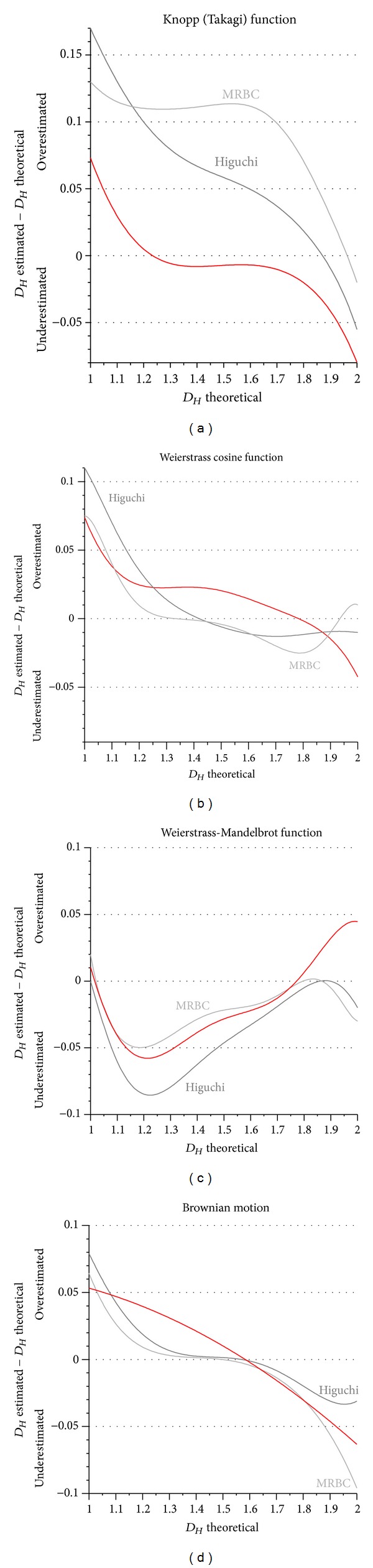
Accuracy of the new *amplitude spectrum fractal dimension method* (MAFDM; red curves; *m* = 1) compared to Higuchi's method and the MRBC method, tested with the following *continuous nowhere differentiable functions* (CNDFs): Knopp/Takagi function (a); Weierstrass cosine function (b); Weierstrass-Mandelbrot function (c); Brownian motion (d).

*D*_MB_ = *D*_*H*_ if the fractal is strictly self-similar. *b* was set to 2, *k* was limited to 50, and *K*(*t*, *D*_MB_) was calculated for one second at a frequency of 1 kHz. The results are shown in Figure 7(a). For the proposed algorithm of the MAFDM, *D*_*Hm*_ is over/underestimated at small (close to 1) and large (close to 2) theoretical *D*_*H*_. The MAFDM is more accurate than Higuchi's and MRBC methods, except for *D*_*H*_ ≥ 1.9.

(*2) Weierstrass Cosine Function W*(*t*, *H*):

(33) $$\begin{array}{c} W\left(t,H\right)=\overset{\infty }{\underset{k=0}{\sum }}\gamma^{-kH}\cos{\left(2\pi \gamma^{k}t\right)}^{k}, \end{array}$$

where *γ* > 1 and *H* is the Hurst exponent ( = 2 − *D*_*H*_). *γ* was set to 5, *k* was limited to 50, and *W*(*t*, *H*) was calculated for one second at a frequency of 1 kHz. The results are shown in Figure 7(b). At medium *D*_*H*_, the MAFDM is marginally less accurate (maximally by 2.5% of the theoretical *D*_*H*_) than Higuchi's and MRBC. 

(*3) Weierstrass-Mandelbrot Function Wm*(*t*, *H*):

(34) $$\begin{array}{c} Wm\left(t,H\right)=\overset{\infty }{\underset{k=-\infty }{\sum }}\frac{1-\cos\left(b^{k}t\right)}{b^{Hk}}. \end{array}$$

For comparative reasons, *b* was set to 1.5, *k* was limited to 50, and *Wm*(*t*, *H*) was calculated for one second at a frequency of 1 kHz. The results are shown in Figure 7(c). The MAFDM has the same accuracy as the MRBC method and performs better than Higuchi's method. 

(*4*)*  Brownian Motion Function*. The time series was calculated in Matlab R2010b (by MathWorks, Natick, MA, USA) with the command *wbfm*(*H*, *n*), where *H* is the Hurst exponent ( = 2 − *D*_*H*_) and *n* is the number of data generated. As the Brownian motion function is stochastic, the shapes of the curves are not identical, and therefore ten different fractal curves were tested for each *D*_*H*_ (from 1 to 2, in 0.1 increments). The results are shown in Figure 7(d). The MAFDM has roughly the same performance as the MRBC method and Higuchi's method.

**Figure 8:**
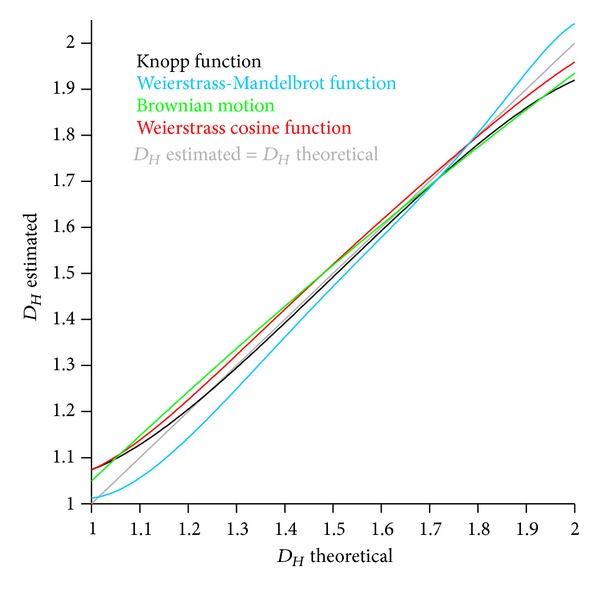
Summary of the accuracy of the *modified amplitude fractal dimension method* (MAFDM; *m* = 1) tested with the four CNDFs (*nowhere differentiable functions*).

**Figure 9:**
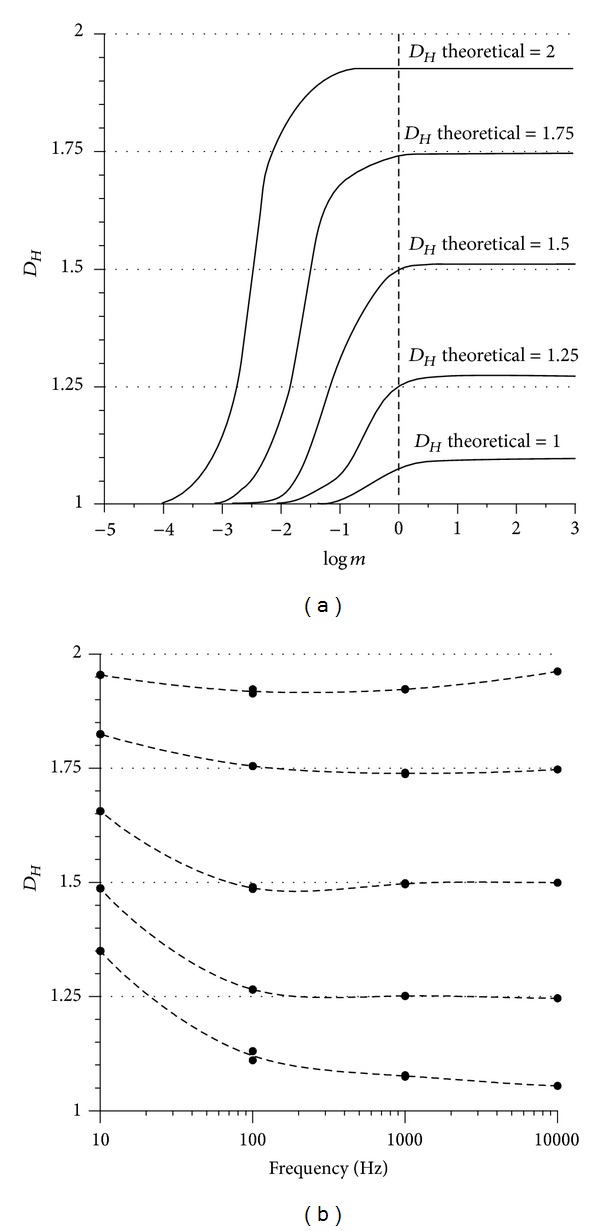
(a) *D*_*Hm*_ of Knopp function against log *m*; (b) *D*_*H*_ of Knopp function against sampling frequency at different number of samples [window width *ww*]; 10 Hz:*ww* = 100, 1000, 10000 data; 100 Hz:*ww* = 100, 1000 data; 1000 Hz:*ww* = 1000, 10000 data; 10000 Hz:*ww* = 10000 data.

In summary (Figure 8), the MAFDM did not show any disadvantage compared to the MRBC method and Higuchi's method, delivered comparable results, and is therefore considered sufficiently accurate. The multiplier *m* was set to 1 in order to compare *D*_*Hm*_ of the unscaled signal to *D*_*Ho*_ of Higuchi's method. *D*_*Hm*_, however, is *m* dependent (Figure 9(a)), and the *D*_*Hm*_ values at *m* = 1 are at the beginning of the right-hand asymptotic segment. The sampling frequency of CNDFs influences the accuracy as well (Figure 9(b)); the higher the frequency is, the closer the theoretical *D*_*H*_ to the estimated one is. In contrast to that, *D*_*Hm*_ is relatively insensitive to window widths (Figure 9(b)). The *D*_*Hm*_ data obtained from the 10 Hz signal are generally higher than the ones of the 100 Hz signal. The reason for this is that the deterministic CNDFs achieve a higher *D*_*Hm*_ by increasing the amplitude of their higher harmonics. The smaller the sampling frequency, the more irregular (“chaotic”) is the signal and the higher is the estimated *D*_*Hm*_.

## 7. Optimisation and Customisation Method

The optimisation method serves for improved decision making. For example, for automated distinction between a physiological signal and a pathological one (possibly due to a life-threatening condition), their *D*_*Hm*_ must not overlap, that is, must not result in false positive or false negative diagnoses. Therefore, the *D*_*Hm*_ of the physiological signal must be as small as possible and the one of the pathological on as large as possible (or vice versa). This is achieved by optimising the amplitude multiplier *m*. The optimal multiplier *m* is neither selected arbitrarily nor subjectively but rather follows engineering optimisation methods, namely,

- maximising the difference between maximal and minimal *D*_*Hm*_ of signals or parts thereof;
- maximising the difference between maximal and average *D*_*Hm*_;
- maximising the ratio of maximal to average *D*_*Hm*_.

**Figure 10:**
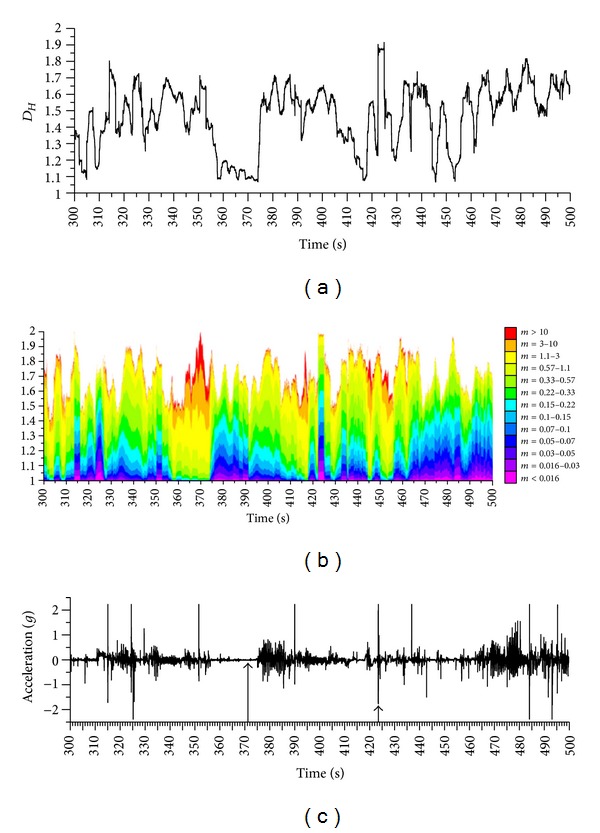
Acceleration of a wheelchair recorded at 60 Hz during a wheelchair rugby match (data from [15]) against time (c); the two arrows refer to the time data with the lowest and highest *D*_*H*_ used for determining the amplitude spectrum of Figure 11; (b) fractal dimension *D*_*Hm*_ (running average with a window width of 151 data points over 2.5 s) of the acceleration signal calculated for different amplitude multipliers *m* (cf. Figure 11); note that the fractal dimension at *m* → *∞* corresponds to the one calculated with Higuchi's method in Figure 2; (a) optimised *D*_*Hm*_ at *m* = 0.8.

Maximally separating the *D*_*Hm*_ of physiological and pathological signals is achieved by plotting the *m*-dependent *D*_*Hm*_ spectra of both signals, and by identifying the maximal *D*_*Hm*_ differential,

(35) $$\begin{array}{c} \Delta D_{Hm}=D_{Hm}\max-D_{Hm}\min, \end{array}$$

where *D*_*Hm*_min⁡ and *D*_*Hm*_max⁡ are the average *D*_*Hm*_ of the physiological and the pathological signals, respectively, if the pathological signal is expected to have the higher *D*_*Hm*_, which anyway results from the spectra. For identification of events within a signal, it depends on the number of events and whether they are relatively evenly distributed. If there are more than two different events and none of them is extremely rare, then those parts of the signal that are expected to have the smallest and largest *D*_*Hm*_ are identified and their Δ*D*_*Hm*_ is maximised according to (35). This is exemplified in Figure 10, in the same signal shown in Figure 2.

**Figure 11:**
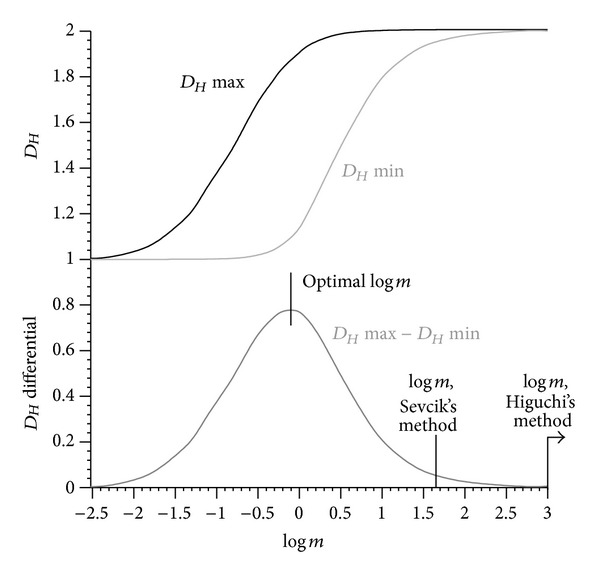
Fractal dimension *D*_*Hm*_ against the amplitude multiplier *m*; *D*_*Hm*_max⁡ = *D*_*Hm*_ with the highest value (at 371.1 s ±75 data of Figure 10, window width of 2.5 s = 151  data); *D*_*Hm*_min⁡ = *D*_*Hm*_ with the smallest value (at 423.5 s ± 75 data of Figure 10); *D*_*Hm*_max⁡−*D*_*Hm*_min⁡ = Δ*D*_*Hm*_, the *D*_*Hm*_ differential; optimal amplitude multiplier *m* is defined at the peak of the *D*_*Hm*_ differential (maximal *D*_*Hm*_ distance between *D*_*Hm*_max⁡ and *D*_*Hm*_min⁡).

Figure 10 shows the spectrum across different *m* values against the time, as well as the events that were considered to have minimal and maximal *D*_*Hm*_. The maximal Δ*D*_*Hm*_ is identified from Figure 11, where minimal and maximal *D*_*Hm*_ were plotted against the logarithm of *m* and the optimised value of *m* was determined from maximal Δ*D*_*Hm*_. At *m* → 0 and *m* → *∞*, both the highest and smallest *D*_*H*_ asymptote to one and two, respectively. The maximal *D*_*Hm*_ range amounts to Δ*D*_*Hm*_ = 0.78. In this specific case, it turned out that the optimal *m* is close to 1, that is, *m* = 0.8, which means that the original signal is coincidentally close to the optimised one. The unit of the unnormalised acceleration signal is *g* (9.81 m/s^2^). If the signal was recorded in m/s^2^, then *m* would have been 0.08, and Δ*D*_*Hm*_ at *m* = 0.8 would have been reduced to one-third of Δ*D*_*Hm*_max⁡. When transforming the signal into a unit square, that is, using Sevcik's unit square method [14], the multiplier of the normalised *y*-amplitude would have been *m* = 43.3 (log⁡*m* = 1.64, Figure 11, amplitude range 4.62*g*, time period 200 s, *m* = 200/4.62), and Δ*D*_*Hm*_ would have been reduced to 6.5% of Δ*D*_*Hm*_max⁡. When using Higuchi's method [7], the multiplier would have been *m* > 1000, and Δ*D*_*Ho*_ would have been reduced to zero (Figures 10 and 11). The multiplier *m* of Higuchi's method results from not calculating the Euclidean distance and thereby reducing the normalised time dimension to zero, which in turn causes *m* → *∞*. The multiplier *m* of Sevcik's method results from transforming the signal to a unit square, thereby stretching the normalised amplitude axis with respect to the normalised time axis.

Plotting the *D*_*Hm*_ values at different *m* against time reveals the influence of *m* on the magnitude of *D*_*Hm*_. In Figure 10, *D*_*Hm*_ coloured in lime corresponds to the maximal *D*_*Hm*_ range. For *D*_*Hm*_ coloured in red (maximal *D*_*Hm*_ across the signal) or purple (minimal *D*_*Hm*_), the range approaches zero and is therefore not ideal for distinguishing different events. This is due to the fact that the highest and smallest *D*_*Hm*_ of this specific signal asymptote to one and two, respectively. At this point, it has to be noted that, whereas *D*_*Hm*_ values at *m* → 0 always asymptote to one, *D*_*Hm*_ values at *m* → *∞* asymptote to *D*_*Ho*_, the fractal dimension of the original unmodified signal. Therefore, the asymptotic value of *D*_*Hm*_max⁡ at *m* → *∞* is not necessarily larger than the one of *D*_*Hm*_min⁡ (as will be shown in Section 8). Before reaching their asymptotic values, *D*_*Hm*_max⁡>*D*_*Hm*_min⁡, but at larger *m*, they can converge and even switch their position. This complicates the usage of Higuchi's method for obtaining clearly separated data of different signals (or parts thereof), as shown in Figures 2 and 10.

If there are only two different events (e.g., physiological and pathological) embedded in a signal, with the latter being rare, then the average *D*_*Hm*_ of the entire signal, *D*_*Hm*_avg, is compared to *D*_*Hm*_ of the rare event (*D*_*Hm*_max⁡ if the rare event produces a higher *D*_*Hm*_ than the rest of the signal). *D*_*Hm*_ of the rare event should not significantly influence the average *D*_*Hm*_. Δ*D*_*Hm*_ then results from

(36) $$\begin{array}{c} \Delta D_{Hm}=D_{Hm}\max-D_{Hm}\text{avg}. \end{array}$$

In order to suppress *D*_*Hm*_avg further, thereby accentuating *D*_*Hm*_max⁡, *D*_*Hm*_ can be optimised as to the *D*_*Hm*_ ratio as follows:

(37) $$\begin{array}{c} D_{Hm}\text{ratio}=\frac{D_{Hm}\max-1}{D_{Hm}\text{avg}-1}. \end{array}$$

The three different optimisation methods, (35)–(37) facilitate that the *D*_*Hm*_ of the different signals are clearly separated by applying a zooming effect to the signals. It will be shown in Section 8, that this zooming effect also provides a filter effect. 

The basic principle of optimising the amplitude of signals is to find the largest possible difference between *D*_*Hm*_ of different signals or parts thereof. The optimisation methods are exemplified by, and explained in, four different cases in Section 8. The *D*_*Hm*_ is calculated with the MAFDM in all four cases.

## 8. Results

**Case 1 (assessment of emotional reactions with EEG)**

**Figure 12:**
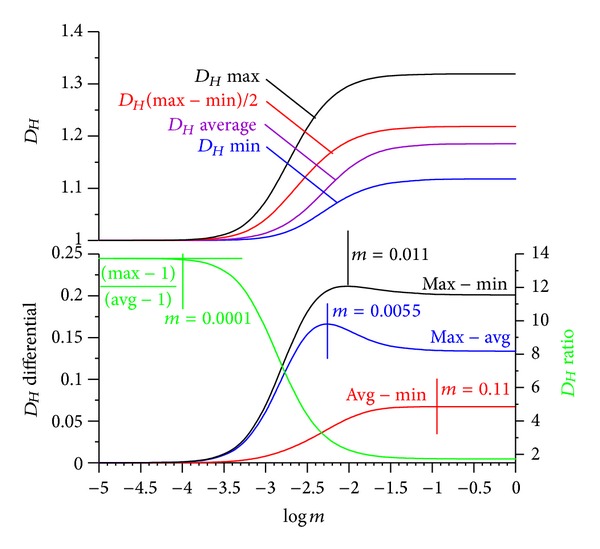
Fractal dimension *D*_*Hm*_ against the amplitude multiplier *m*; *D*_*Hm*_max⁡ = *D*_*Hm*_ with the highest value (at 24.2 s ± 127 data of Figure 13, window width of 1 s = 255  data); *D*_*Hm*_min⁡ = *D*_*Hm*_ with the smallest value (at 5.5 s ± 127 data of Figure 13); *D*_*Hm*_ average (avg) = *D*_*H*_ averaged over 60 s; max⁡−min⁡, max⁡−avg, and avg − min⁡ = *D*_*Hm*_ differentials; (max⁡−1)/(avg − 1) = *D*_*Hm*_ ratio.

**Figure 13:**
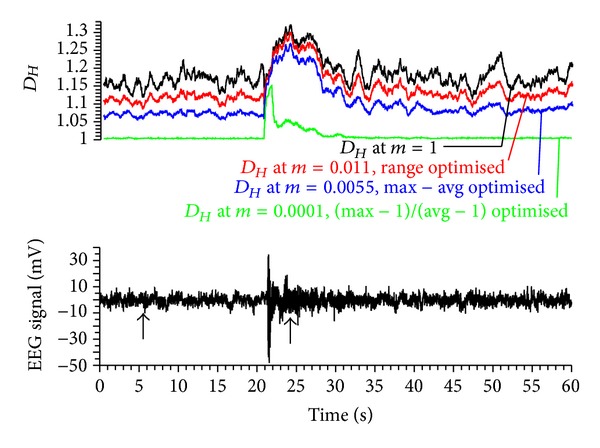
EEG signal recorded at 256 Hz against time (bottom subfigure); the two arrows refer to the time data with the lowest and highest *D*_*H*_ used for determining the amplitude spectrum of Figure 12; top subfigure: fractal dimension *D*_*Hm*_ (running average with a window width of 255 data points over 1 s) of the EEG signal calculated for different amplitude multipliers *m* (cf. Figure 12).

Fuss and Kulish (unpublished data) recorded the EEG of test persons during watching short movies with unexpected scary events (the so-called prank videos or screamers) in order to measure the intensity of the emotional pressure. By quickly plotting the *D*_*Hm*_ at different *m*, it becomes evident that the *D*_*Hm*_ correlates with the emotional pressure during the aftermath of startling. It is therefore advisable to suppress the magnitude of the small *D*_*Hm*_, by keeping the one of the maximal *D*_*Hm*_. The aim is therefore to maximise the *D*_*Hm*_ range between the highest *D*_*Hm*_ and the average *D*_*Hm*_ (Figure 12). The average *D*_*Hm*_ is calculated across the entire signal and is, in this example, smaller than the average of the highest and smallest *D*_*Hm*_. This can be achieved by maximising the *D*_*Hm*_ differential of highest *D*_*Hm*_, and average *D*_*Hm*_ whereas keeping the *D*_*Hm*_ differential of average *D*_*Hm*_ and smallest *D*_*Hm*_ as small as possible. Alternatively, the *D*_*Hm*_ ratio of highest *D*_*Hm*_ minus 1 to average *D*_*Hm*_ minus 1 can be calculated according to (37).  Figure 13 shows the *D*_*Hm*_ at the original signal amplitude, the range optimised *D*_*Hm*_ and the *D*_*Hm*_ optimised to the maximal range between the highest *D*_*Hm*_ and the average *D*_*Hm*_. The effect of the latter is that the magnitude of the highest peak is kept, whereas the magnitude of smaller *D*_*Hm*_ is reduced. A further effect is that the noise level of the *D*_*Hm*_ decreases, specifically at small *D*_*Hm*_. The optimisation method therefore provides an additional filter effect. The *D*_*Hm*_ ratio increases as *m* decreases and finally asymptotes at small *m*. The optimal *m* is located at the beginning of the asymptotic segment: *m* = 0.001. The maximal *D*_*Hm*_ at this multiplier is no longer located at 24.2 s (Figure 13) but rather at 21.9 s, where the EEG signal has its maximal amplitude. Also the *D*_*Hm*_ noise before and after startling is reduced to a minimum.

**Case 2 (comparison of the same signal recorded with different sensors)**

**Figure 14:**
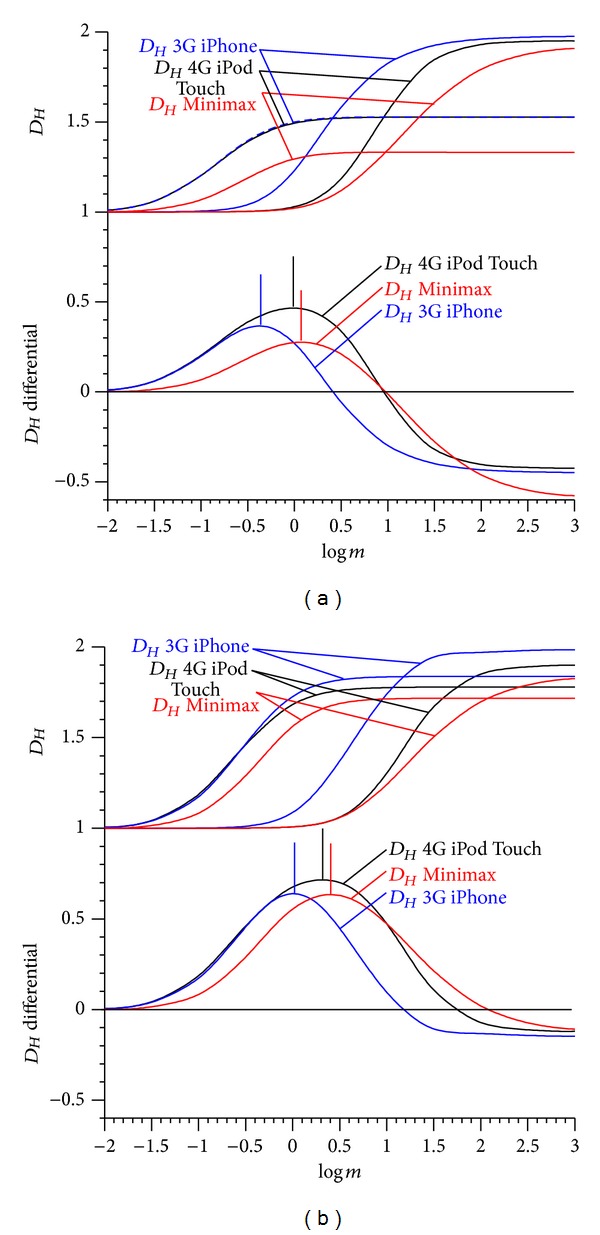
Fractal dimension *D*_*Hm*_ against the amplitude multiplier *m*; *D*_*Hm*_  differential = *D*_*Hm*_max⁡−*D*_*Hm*_min⁡; left subfigure: original signal at 100 Hz; right subfigure: frequency reduced to 50 Hz.

**Figure 15:**
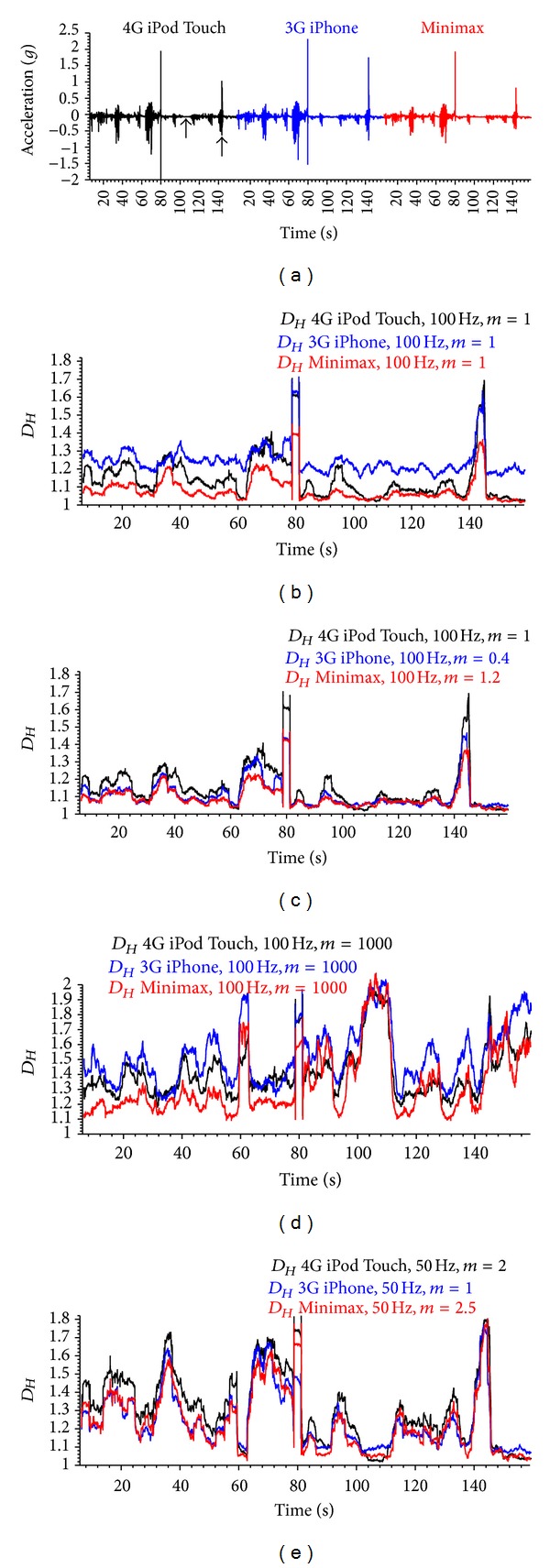
Wheelchair acceleration signal recorded with three different devices at 100 Hz against time (a); the two arrows refer to the time data with the lowest and highest *D*_*Hm*_ values used for determining the amplitude spectrum of Figure 14; (b): unoptimised fractal dimension *D*_*Hm*_ of the 100 Hz signal (*m* = 1, running average with a window width of 251 data points over 2.5 s); (c): optimised fractal dimension *D*_*Hm*_ of the 100 Hz signal; (d): fractal dimension *D*_*Ho*_ of the original 100 Hz signal at *m* = 1000; (e): optimised fractal dimension *D*_*Hm*_ of the signal reduced to 50 Hz (running average with a window width of 125 data points over 2.5 s) calculated for different amplitude multipliers *m* (cf. Figure 14 and Table 1).

Fuss and Chua (unpublished data) investigated the sensors influence on *D*_*Hm*_. They recorded the acceleration signal of a rugby wheelchair at different activities (collisions, pushing, coast down, and zero activity) at 100 Hz with three different sensors mounted on the frame of the chair (3G Apple iPhone, 4G Apple iPod Touch, and Minimax by Catapult). For the smart phones, it is evident that the generation number (3G and 4G) was decisive and not whether the device was an iPhone or an iPod Touch. Subsequently, the signal was reduced to 50 Hz by taking every other point. Both signals were range optimised (Figures 14 and 15), the results of which are shown in Table 1.

The unoptimised *D*_*Hm*_ of the 100 Hz signals (Figure 15(b)) showed pronounced differences between the *D*_*Hm*_ values. This could be due to the different device resolutions: 3G iPhone: 0.018112*g*, Minimax: 0.006*g*, and 4G iPod Touch: 0.000015*g*. The high resolution of the iPod Touch could be a result of data averaging or filtering. The *D*_*Hm*_ of the 3G iPhone signal is higher, on average, compared to the two other signals. After optimisation, the *D*_*Hm*_ of the 3G iPhone signal dropped and fell within the range of the *D*_*Hm*_ of other two signals. Reducing the frequency to one half merely increases *D*_*Hm*_ but does not markedly change the trend.

Furthermore, the *D*_*Hm*_ optimisation diagram (Figure 14) shows an interesting result: the two *m*-spectrum curves intersect, as the *D*_*Hm*_ of minimal activity at maximal *m* is higher than the one of maximal activity at maximal *m*. After the intersection point, the *D*_*Hm*_ difference is negative. This does not only explain the high values of Higuchi's method (Figure 15(d)) at near-zero values of the acceleration signal, but also does this justify the optimisation procedure: select those parts of the signal which should provide different *D*_*Hm*_ and increase the *D*_*Hm*_ differential to a maximum through optimisation. Higuchi's method would have been useless in this specific case (Figure 15(d); *m* = 1000), as collisions and low activity would have produced *D*_*Hm*_ in the same range, and the noise would have also been more pronounced. In fact, it reflects the result of Higuchi's method shown in Figures 2 and 10, namely, that Higuchi's method produces maximal *D*_*Hm*_ in acceleration signals of low-level activity.

**Case 3 (acoustic signature of a ball impact)**

**Figure 16:**
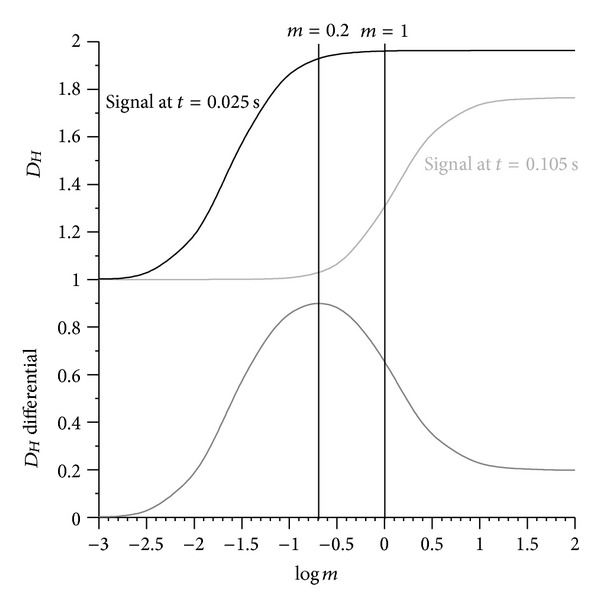
Fractal dimension *D*_*Hm*_ against the amplitude multiplier *m*.

**Figure 17:**
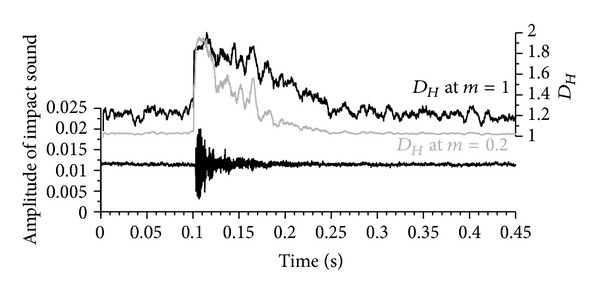
Acoustic signal recorded at 11.025 kHz against time (bottom subfigure); top subfigure: fractal dimension *D*_*Hm*_ (running average with a window width of 55 data points over 5 ms) of the acoustic signal calculated for different amplitude multipliers *m* (cf.  Figure 16).

Fuss [20] recorded the impact sound of golf balls at 11.025 kHz and correlated it to the hardness of the balls. In addition to FFT (for determining the power spectrum and the frequencies of the impact sound), the fractal dimension of the impact sound can be calculated.  Figure 16 shows the range optimisation diagram with the optimal *m* at 0.2. Reducing the original amplitude to one-fifth results in a smoother signal, spanning a larger *D*_*Hm*_ range (Figure 17). Again, the filter effect is apparent.

**Case 4 (quality of noise)**

**Figure 18:**
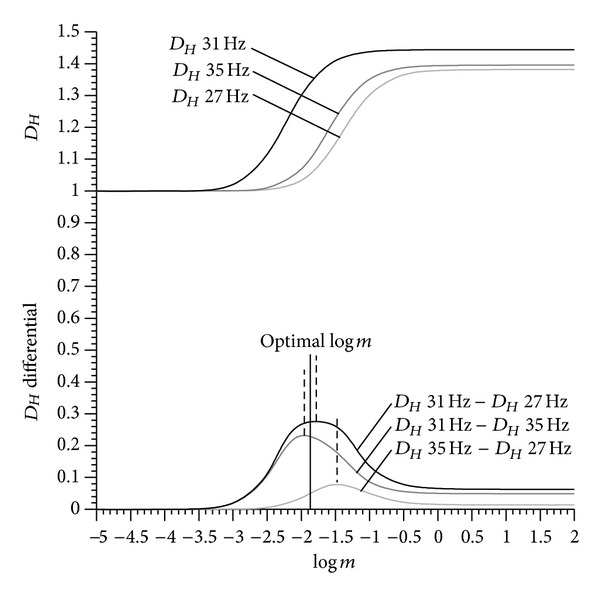
Fractal dimension *D*_*Hm*_ (calculated from the mean of the running average) of the three signals against the amplitude multiplier *m*.

Fard, Subic, Lo and Fuss (unpublished data, submitted) recorded the acoustic sound pressure levels of a car seat at different excitation frequencies (27, 31, and 35 Hz). The *D*_*Hm*_ of the acoustic signal of the rattling seat suggested that the frequency of 31 Hz produces the highest *D*_*Hm*_, whereas the *D*_*Hm*_ values of the signal after 27 and 35 Hz excitation were almost identical. The difference in *D*_*Hm*_ between the three signals was optimized, and the average *m*-value of the maximal range between the 31 Hz excitation and 27&35 Hz excitations was selected (Figure 18). For comparative reasons, *m* must be the same for all three signals. Even before optimisation, the *D*_*Hm*_ values of the three excitation frequencies were significantly different at *P* < 0.05 (due to the high recording frequency of 10 kHz).

**Figure 19:**
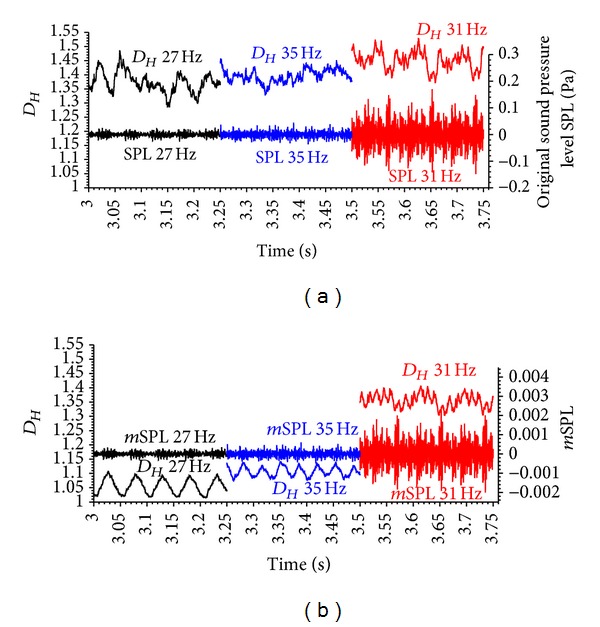
Acoustic signals and their corresponding fractal dimensions *D*_*Hm*_ (running average with a window width of 201 data points over 20 ms) recorded at 10 kHz against time; SPL: sound pressure level in Pascal; *m*SPL: SPL multiplied by amplitude multiplier *m*; (a) fractal dimensions *D*_*Hm*_ of the acoustic signal calculated from original signals (amplitude multiplier *m* = 1; *D*_*Hm*_ *≈D*_*Ho*_) with mean *D*_*Hm*_ values of 27 Hz: 1.377, 35 Hz: 1.394, and 31 Hz: 1.441; (b) fractal dimensions *D*_*Hm*_ calculated from amplitude multiplier *m* = 0.0135 (cf. Figure 18) with mean *D*_*Hm*_ values of 27 Hz: 1.057, 35 Hz: 1.109, and 31 Hz: 1.348.

Figure 19 shows the *D*_*Hm*_ of the original signal (*m* = 1) and the optimised signal amplitude (*m* = 0.0135). Signal amplitude optimisation not only separates the *D*_*Hm*_ levels of the three different signals but also clearly exhibits a filter effect. *D*_*Hm*_ at the original signal amplitude suggests a periodic change of *D*_*Hm*_ only in the 31 Hz excitation signal, whereas the 27 and 35 Hz excitations signals are far from periodic and rather noisy and chaotic in nature. After applying the optimised *m* value, all signals are clearly periodic.

## 9. Discussion

This paper introduces a method for optimising the fractal dimension *D*_*Hm*_ of a signal for improved decision making by scaling the signal amplitude after normalising amplitude and time axes to dimensionless values, such that the Euclidean distance between consecutive data points can be calculated. Scaling time series signals for calculating *D*_*Hm*_ is not new. 

(1) Scaling was applied by Sevcik [14] when normalising the signal to the amplitude range and maximal time period, thereby transforming the signal into a unit square, such that amplitude and time axes have the same length of 1. This scaling method is chosen arbitrarily and is therefore a subjective method. The fractal dimension *D*_*H*_ of this transformed signal is only applicable to its unit square, but *D*_*H*_ of signals with different amplitude ranges and/or different time periods cannot be compared any more due to different amplitude multipliers (m = time period/amplitude range).

(2) Scaling is applied by mono-dimensional methods such as Higuchi's method [7]. As the time dimension of the signal is not considered, the signal is transformed into a vertically oriented rectangle with horizontal side length of zero, thereby transforming the signal into a vertical line. The same effect is achieved by multiplying the amplitude axis of the signal by a very large number (*m* → *∞*) such that the time axis becomes comparatively infinitesimally small and approaches zero.

As there is no rule for scaling—otherwise Sevcik's and Higuchi's methods would stand in contrast to each other—any scaling method can be applied, provided that it is neither arbitrary nor subjective. The method introduced in this paper follows an engineering optimisation approach, by maximising the difference between the smallest and largest *D*_*Hm*_ values across signals to be compared, or across parts of a signal. This is achieved by applying an optimised multiplier *m* to the normalised amplitude of the signals, and this multiplier must be the same in all signals (or parts thereof) to be compared. In most cases, Higuchi's method narrows down the difference between the smallest and largest *D*_*Hm*_ values and therefore is not suitable for decision making. In most cases, the optimal multiplier *m* of the normalised amplitude *y* is smaller than 1 (depending on the original unit if the recorded data); the smaller *m* is, the less noisy the *D*_*Hm*_ dataset is (obtained from a sliding window method) and the more regular the *D*_*Hm*_ of periodic signals is due to the filter effect of small *m*-values. The proposed optimisation technique enables the researcher to customise the normalised signal amplitude such that maximal information is obtained, specifically for improved decision making and this with an additional filtering option.

It is suggested that the normalised amplitude *y*, and not the normalised time *x*, is scaled by *m*. Increasing the normalised frequency *f*_0_ by a factor of *m*  ( = reducing  Δ*x*/*m*) delivers the same *D*_*Hm*_ as increasing the amplitude *y* by a factor of *m*. This explains the effect of Higuchi's method, which does not calculate the Euclidean distance between data points and is therefore unaffected by Δ*x*. Reducing Δ*x* to zero means that *m* → *∞*. Therefore, Higuchi's method delivers the asymptotic value on the right side of the range spectrum, at maximal *m* (Figures 11 and 14, with the effect seen in Figures 2, 10, and 15(d)). In fact, *D*_*Ho*_ calculated from Higuchi's method is identical to the maximal *D*_*Hm*_ in Figure 10. Scaling the frequency *f*_0_ via *m* instead of the amplitude, however, is not advisable, as *f*_0_ is connected to the actual sampling frequency of the measurement device. 

It is evident that the multiplier *m* of the normalised signal amplitude must be the same in all related signals for comparative reasons. For example, this applies to parts of a signal which are compared, as well as to signal data related to a therapeutic period (of one or more patients) or to a specific pathology across a cohort of patients. This is not the case in Sevcik's unit square method, as different multipliers result if the amplitude range and/or time periods of signals to be compared are not identical. If *D*_*Hm*_ is determined as a function of normalised time with a running average analysis, then the window width has to be taken care of. If the window width is widened, then the *D*_*Hm*_ of the signal is less noisy, the valleys become narrower (and the peaks wider) along the time axis, and the valleys become shallower (and the peaks flatter) along the amplitude axis. The optimal multiplier *m* has to be determined at the same window width (e.g., Figure 18) as subsequently used for the optimised fractal dimension analysis (e.g., Figure 19). For the optimisation procedure, the *D*_*Hm*_ averaged over time or over the window width is used (e.g., Figure 18). Different recording devices are likely to deliver different *D*_*Hm*_ if their sensor specifications do not match. Optimisation of the *D*_*Hm*_ signal can mitigate the problem. 

Finally, it has to be pointed out again that the fractal dimension *D*_*Hm*_, obtained after modifying the signal by multiplying its amplitude by the multiplier *m*, is no longer the fractal dimension *D*_*Ho*_ of the original signal but rather the fractal dimension *D*_*Hm*_ of the modified or transformed signal. *D*_*Hm*_ serves only for comparing two or more signals (or parts of the same signal) for the purpose of decision making and for classifying or quantifying the intensity of events occurring throughout the time series signal. Thus, *D*_*Hm*_ does not serve for accurate evaluation of the fractal dimension *D*_*Ho*_ of the original signal. Yet, fractal dimensions of signals are often compared in order to draw conclusions, the practical applications of which ultimately result in decision making. The latter is optimised with the method described and presented in this study.

## 10. Conclusions 

Different, usually well-performing, methods for computing the fractal dimensions of time series can produce opposite results in extreme signals, namely, dimensions of 1 (or even <1) and 2. These results can be reproduced when calculating the Euclidean distance between data points and *modifying* their amplitude by a multiplier. Equally, standard methods for computing fractal dimensions use different scaling methods, that is, different amplitude multipliers, which makes it difficult to compare fractal dimensions obtained from different methods.

In order to overcome this problem, a fractal dimension optimisation method is proposed that computes the fractal dimension of a normalised (dimensionless) and modified time series signal with a robust algorithm and a running average method, and maximises the difference between two fractal dimensions, for example, a minimum and a maximum one.

The optimisation method developed in this study is essential for decision making, particularly when the decision hinges on the condition that two different signals (or parts of a signal) have different fractal dimensions. The optimisation method is applied to calculating the maximal fractal dimension differential, as a function of the amplitude multiplier. Alternatively, the fractal dimension differential between maximal dimension and average dimension can be maximised. This optimisation method enhances fractal dimensions above average and suppresses those below average. Maximising the ratio of maximal to average dimension provides a further optimisation effect. 

The smaller the amplitude multiplier, the stronger is the filter effect on the fractal dimension. Large multipliers result in a very noisy fractal dimension signal. The filter effect even reveals periodic events of a signal and enhances larger fractal dimensions. 

The fractal dimension *D*_*Hm*_, obtained after modifying the signal by multiplying its amplitude by the multiplier *m*, is no longer the fractal dimension *D*_*Ho*_ of the original signal but rather the fractal dimension *D*_*Hm*_ of the modified signal only, used solely for the purpose of decision making and for classifying or quantifying the intensity of events occurring throughout the time series signal.

## Figures and Tables

**Table 1:** Optimal multipliers *m* of the normalised acceleration amplitude of the three different devices tested.

	Optimal *m* at 100 Hz	Optimal *m* at 50 Hz
4G iPod Touch	1.0	2.0
3G iPhone	0.4	1.0
Minimax	1.2	2.5

## References

[1] Takayasu H (1990). *In the Physical Sciences*.

[2] Hausdorff F (1919). Dimension und äußeres Maß. *Mathematische Annalen*.

[3] Carathéodory C (1914). Über das lineare Maß von Punktmengen—eine Verallgemeinerung des Längenbegriffs. *Nachrichten von der Gesellschaft der Wissenschaften zu Göttingen, Mathematisch-Physikalische Klasse*.

[4] Richardson LF (1961). The problem of contiguity: an appendix of statistics of deadly quarrels. *General Systems Yearbook*.

[5] Mandelbrot BB (1967). How long is the coast of Britain? Statistical self-similarity and fractional dimension. *Science*.

[6] Katz MJ (1988). Fractals and the analysis of waveforms. *Computers in Biology and Medicine*.

[7] Higuchi T (1988). Approach to an irregular time series on the basis of the fractal theory. *Physica D*.

[8] Sevcik C (2006). On fractal dimension of waveforms. *Chaos, Solitons and Fractals*.

[9] Raghavendra BS, Dutt DN (2010). Computing fractal dimension of signals using multiresolution box-counting method. *International Journal of Engineering and Mathematical Sciences*.

[10] Raghavendra BS, Narayana Dutt D (2009). A note on fractal dimensions of biomedical waveforms. *Computers in Biology and Medicine*.

[11] Castiglioni P (2010). What is wrong in Katz’s method? Comments on: ‘a note on fractal dimensions of biomedical waveforms’. *Computers in Biology and Medicine*.

[12] Mandelbrot BB (1983). *The Fractal Geometry of Nature*.

[13] Blaszczyk JW, Klonowski W (2001). Postural stability and fractal dynamics. *Acta Neurobiologiae Experimentalis*.

[14] Sevcik C (1998). A procedure to estimate the fractal dimension of waveforms. *Complexity International*.

[15] Fuss FK, Subic A, Chua JCC (2012). Analysis of wheelchair rugby accelerations with fractal dimensions. *Procedia Engineering*.

[16] Esteller R, Vachtsevanos G, Echauz J, Litt B (2001). A Comparison of waveform fractal dimension algorithms. *IEEE Transactions on Circuits and Systems I*.

[17] Kairies H-H (1997). Functional equations for peculiar functions. *Aequationes Mathematicae*.

[18] Kannappan P (2009). Functional equations and inequalities with applications. *Springer Monographs in Mathematics*.

[19] Kulish V, Sourin A, Sourina O (2006). Human electroencephalograms seen as fractal time series: mathematical analysis and visualization. *Computers in Biology and Medicine*.

[20] Fuss FK, Fuss FK, Subic A, Ujihashi S (2008). Non-linear viscoelastic properties of golf balls. *The Impact of Technology on Sport II*.

